# Prevalence and correlates of nonsuicidal self-injury among youths in Singapore: findings from the National Youth Mental Health Study

**DOI:** 10.1186/s13034-025-00885-6

**Published:** 2025-03-21

**Authors:** Sherilyn Chang, Janhavi Ajit Vaingankar, Bernard Tan, Yeow Wee Brian Tan, Ellaisha Samari, S. Archana, Yi Chian Chua, Yi Ping Lee, Charmaine Tang, Swapna Verma, Mythily Subramaniam

**Affiliations:** 1 Research Division, Institute of Mental Health, Singapore, Singapore; 2 Department of Psychosis, Institute of Mental Health, Singapore, Singapore; 3 DUKE-NUS Medical School, Singapore, Singapore; 4 Saw Swee Hock School of Public Health, National University Singapore, Singapore, Singapore; 5 Lee Kong Chian School of Medicine, Nanyang Technological University, Singapore, Singapore

**Keywords:** Nonsuicidal self-injury, NSSI, Deliberate self-harm inventory, Self-harm, Youth

## Abstract

**Background:**

Nonsuicidal self-injury (NSSI) is a common phenomenon; a recent meta-analysis of studies conducted among non-clinical adolescents reported a global lifetime prevalence rate of 22.0%. NSSI results in significant impairment and is associated with negative outcomes later in young adulthood. There is, however, a dearth of research on the occurrence of NSSI in Singapore’s youth population. Past studies examining NSSI behaviours among youths in Singapore were conducted using clinical samples, which tend to report a higher prevalence compared to community samples. The present study aims to establish the prevalence of NSSI and examine its associated sociodemographic and psychosocial correlates in the general youth population.

**Methods:**

This study included 2600 youths aged 15–35 years who participated in the National Youth Mental Health Study, a nationwide cross-sectional survey of the mental health status of youths in Singapore. The Deliberate Self-Harm Inventory and Depression Anxiety Stress Scales Short Form were used to assess NSSI behaviours and mental health symptoms. Data on coping strategies, perceived social support and resilience were also collected.

**Results:**

The lifetime prevalence of NSSI among youths in Singapore was 25.0%, and the 12-month prevalence was found to be 6.8%. The median age of onset for lifetime NSSI was 14 years. Significantly higher odds of lifetime NSSI were observed among youths aged 15–29 years, females, and youths with lower educational attainment. Youths with severe and extremely severe symptoms of depression and anxiety and those with greater use of avoidance coping strategy were associated with higher odds of lifetime NSSI. Higher resilience scores were associated with lower odds of lifetime NSSI.

**Conclusion:**

1 in 4 youths in Singapore had engaged in self-injurious behaviour at least once in their lifetime. Screening and early intervention programs could be targeted at the more vulnerable youth groups such as those in early- and mid- adolescence. Potential areas for future research and interventions could include resilience building and educating youths on adaptive coping strategies. The limitations of the cross-sectional study design and the use of self-reported data should be considered when interpreting the study findings.

## Introduction

Various terminologies have been used to describe self-injurious behaviour, including nonsuicidal self-injury (NSSI). NSSI refers to a range of behaviours that involve the deliberate destruction of one’s body tissue without suicidal intent and for purposes not socially sanctioned [1]. Though there are some inconsistencies and ambiguities over what constitutes as a NSSI behaviour (see Lengel et al. [2] for a discussion on NSSI definitions), commonly observed NSSI behaviours include cutting, hitting, and burning one's own body. It is proposed that the primary function of self-injurious behaviours in adolescents is to regulate emotional or physiological experiences (e.g., to reduce negative emotions, to generate feelings), although it can also serve the purpose of modifying one’s social environment (e.g., to avoid something unpleasant, to gain attention from someone [3]).

The United Nations defines youth as individuals aged 15–24 years; however, this classification can vary across countries and is influenced by sociocultural, institutional, economic, and political factors [4]. Nonetheless, youth is widely recognised as a transitional period encompassing the adolescent years, characterised by profound biological and psychosocial changes. This period is also associated with increased vulnerability to the development of mental health problems and risky behaviours, including NSSI [5]. Globally, the estimated prevalence of NSSI was 5.5% among adults and much higher among adolescents and young adults, with prevalence rates of 17.2% and 13.4% respectively [6]. A recent meta-analysis of studies published between 2010 and 2021 reported a lifetime NSSI prevalence of 22.0% and a 12-month prevalence of 23.2% among non-clinical samples of adolescents worldwide [7]. The youth demographic is critical for the study of NSSI given that studies have shown that the age of onset typically peaks at around 12–15 years [8, 9] and the rates and frequency of NSSI behaviours increase during younger adolescence, which then subsequently decline during older adolescence and young adulthood [10, 11]. There is some evidence to suggest that NSSI has become more prevalent over the years [12], and the occurrence of the COVID-19 pandemic may have contributed to a further increase in the prevalence of self-injurious behaviours [13–15].

Some of the factors associated with a higher risk of NSSI in youths include female gender, mental disorders, adverse childhood experiences, bullying, and problem behaviours such as alcohol use and smoking [16]. Among these variables, female gender, depression, and peer victimization have been found to prospectively predict NSSI in adolescents [17]. A number of studies have also demonstrated a link between body image dissatisfaction and NSSI in youths [18–20]. Other psychosocial factors associated with NSSI in youths include coping strategies, resilience, self-esteem, and social support [21–23].

Results from longitudinal cohort studies have also established the relationship between NSSI in youths and subsequent negative outcomes. Compared with youths without NSSI behaviour, youths who engaged in NSSI were at higher risk of subsequent self-injury, alcohol and/or substance use disorder, and psychiatric inpatient care [24]. Repetitive NSSI behaviour in early adolescence was associated with higher levels of stress, anxiety, NSSI, and emotion regulation difficulties ten years later in young adulthood [25]. The study concluded that stable repetitive NSSI occurring during adolescence increases the risk of mental health issues in young adulthood. Furthermore, NSSI was consistently found to be a predictor of suicidality, above and beyond the effects of depression on suicidal behaviours (see the review conducted by Hamza et al. [26]). These adverse health and psychosocial outcomes associated with NSSI warrant greater attention to examine self-injurious behaviours in youths.

Singapore is a multi-ethnic city-state located in Southeast Asia, with a total resident population (i.e., Citizens and Permanent Residents) of 4.15 million in June 2023, of which one in four were individuals aged 15–34 years [27]. Youth in Singapore is defined as individuals aged between 15 and 35 years [28]. To date, there is no data on NSSI in the general youth population in Singapore. Studies on self-injurious behaviour among youths in Singapore have largely utilized clinical samples which tend to report a higher prevalence than community samples [29]. A study among youth outpatients from a psychiatric hospital found that 58.8% engaged in deliberate self-harm (a more encompassing term to include self-injurious behaviours with or without suicidal intent [30]), and another study using a retrospective review of medical records obtained from adolescent psychiatric outpatients of a general hospital reported a NSSI prevalence of 23.1% [31]. To bridge the gaps in research, the present study was conducted to examine NSSI behaviours in the general youth population in Singapore. Specifically, the study aims to (i) establish the prevalence and age of onset of NSSI in youths; (ii) examine the forms of NSSI behaviours youths engaged in; and (iii) explore sociodemographic correlates and psychosocial factors associated with NSSI.

## Methods

### Study design and sample

Data for this study were obtained from a nationwide survey, the National Youth Mental Health Study (NYMHS), conducted to assess the status of youth’s mental health in Singapore (NYMHS methodology has been described in detail in an earlier article [32]). Briefly, the NYMHS was a cross-sectional epidemiological study of 2600 youths aged 15–35 years residing in Singapore. Inclusion criteria for the study were: (i) being a Singapore Citizen or Permanent Resident; (ii) aged 15–35 years; (iii) literate in English, Mandarin, Bahasa Melayu, or Tamil; and (iv) able to provide written informed consent (with consent from a legally acceptable representative required for those aged below 21 years). Youths who were unable to complete the survey on their own were excluded from the study. Convenience sampling was employed, and youths from various districts in Singapore were approached via household-level sampling using randomly generated postal codes. Individuals who fulfilled the study inclusion criteria were approached at their residences and invited to participate in the survey. Street intercept was also used to contact hard-to-reach groups (e.g., youths in National Service, youths from the younger age groups). Oversampling of youths by age, gender, and ethnicity was performed to obtain an adequate sample size and improve the reliability of estimates when conducting subgroup analyses. Overall, 1948 (76.3%) participants were recruited by household sampling and 652 (23.7%) hard-to-reach youths were recruited via street intercept. Individuals who agreed to take part in the study first provided informed consent during which they were informed of the study purpose and procedures, survey anonymity, and data confidentiality. After written informed consent was obtained, participants were handed a tablet to complete the self-administered survey and in a language they preferred (i.e., English, Chinese, Malay, or Tamil). Data collected on the tablet were de-identified and accessible only to the research team. Each survey took around 1.5 to 2 hours to complete, and participants were reimbursed SGD50 upon completion of the study. Fieldwork for data collection began in October 2022 and was completed in June 2023. The study was approved by the institutional ethics committee (National Healthcare Group Domain Specific Review Board) before the study commenced.

## Measures

### Deliberate self-harm inventory (DSHI; [33])

The DSHI was used to assess NSSI behaviour in this study. It consists of 17 items and collects information on the type of self-injurious behaviour, age of onset, frequency, severity, and duration of the behaviour. Examples of NSSI behaviour examined in the DSHI included cutting, carving words into the skin, dripping acid, punching oneself etc. Item 17 in the DSHI allows an open-ended response to assess any other forms of self-injurious behaviour not already mentioned. Noting that contention remains over which specific acts ought be classified as NSSI [2], an inclusive approach was taken when reviewing responses to Item 17: the item was scored as long as the behaviour described overlaps with the conceptualization of NSSI as nonsuicidal behaviour that results in direct injury to body tissue, regardless of a presence of a wound (i.e., behaviours that did not describe the extent of injury were included, e.g., punching wall, slapping of oneself). Behaviours resulting in indirect harm (e.g., self-poisoning, overdose), with suicidal intent (e.g., jumping off a building, drowning), and describing risky behaviours (e.g., smoking more than usual) were excluded.

### Depression anxiety stress scales short form (DASS-21; [34])

The DASS-21 was used to assess symptoms of depression, anxiety, and stress. It consists of three subscales with seven items in each subscale. Examples of items on the depression subscale include “I felt down-hearted and blue”; the anxiety subscale includes items such as “I felt I was close to panic”; the stress subscale includes “I found it hard to wind down”. Participants were asked to rate the extent to which these items applied to them over the past week on a scale from 0 = *did not apply at all* to 3 = *applied very much or most of the time*. Subscale scores were obtained by summing the items in each subscale and multiplying them by two as per scoring guidelines. Higher scores indicate greater severity of the symptoms, which can be classified into severity categories of normal, mild, moderate, severe and extremely severe [34]. Cut-off scores of ≥ 21 (depression), ≥ 15 (anxiety), and ≥ 26 (stress) were used in this study to identify individuals with severe and extremely severe levels of symptoms. The subscales demonstrated good to excellent internal consistency for the study sample: depression Cronbach’s α = 0.91, anxiety Cronbach’s α = 0.87, and stress Cronbach’s α = 0.89.

### Body Shape Questionnaire-8C version (BSQ-8C; [35])

The BSQ-8C is based on preoccupations of body shape and weight and is used to assess body dissatisfaction in this study. The scale has 8 items scored from 1 = *Never* to 6 = *Always* on statements relating to body shape concerns in the past four weeks. Scores are summed and a higher total score indicates greater body shape dissatisfaction. For the purpose of this study, individuals with a score of 26 and above (indicating moderate to marked body shape concerns) were identified as having significant body dissatisfaction. The scale demonstrated excellent internal consistency in this study sample with a Cronbach’s alpha of α = 0.95.

### Alcohol use disorder identification test (AUDIT; [36])

The AUDIT is a 10-item screening tool developed by the World Health Organization (WHO) to assess alcohol consumption and alcohol-related problems. Scores on the items are summed and the total score ranges from 0 to 40. Following WHO guidelines, scores of 8–14 indicate harmful alcohol consumption, and a score of 15 or more suggests likelihood of alcohol dependence. A cut-off score of 8 and above is used to identify hazardous alcohol use in this study. A good Cronbach’s alpha of α = 0.88 was obtained in this study sample.

### Multidimensional scale of perceived social support (MSPSS; [37])

The MSPSS is a 12-item scale used to measure perceived social support received from three sources: Family, Friends, and a Significant Other. Participants were asked to rate their level of agreement on items such as “I get the emotional help and support I need from my family” on a seven-point scale, from 1 = *strongly disagree* to 7 = *strongly agree*. A mean score was derived by taking the average of the sum of all 12 items, and higher scores indicate greater perceived social support. The scale obtained excellent internal consistency using the study sample (Cronbach’s α = 0.94).

### Connor-Davidson Resilience Scale (CD-RISC; [38])

The 25-item CD-RISC is a widely used instrument to assess resilience and has been used in various study populations. Participants rated the items on a scale from 0 = *not true at all* to 4 = *true nearly all the time* and a total score was obtained by summing item scores. Higher scores reflect higher resilience. An excellent Cronbach’s alpha of α = 0.95 was obtained in this study.

### Coping strategy indicator (CSI; [39])

The CSI consists of 33 items and is used to assess the degree to which three specific coping strategies are utilized in response to stress. Two of these strategies were examined in this study: Avoidance and Problem-Solving. The Seeking Social Support strategy was not included given that perceived social support was examined in this study using the MSPSS. These two variables are likely to be highly correlated, which would result in unreliable estimates if both variables were simultaneously included. Participants were asked to identify a stressful event that occurred and rate statements on coping behaviours in relation to the stressful event on a scale from 1 = *not at all* to 3 = *a lot*. An example of coping behaviours in the Avoidance subscale includes “*Daydreamed about better times*” and an example from the Problem-Solving subscale includes “*Set some goals for yourself to deal with the situation*”. Scores on each subscale were summed, and higher scores indicate greater use of the coping strategy. The Avoidance subscale and Problem-solving subscale demonstrated good internal consistency in this study sample, with Cronbach’s alpha of α = 0.84 and α = 0.89 respectively.

Sociodemographic information including gender, age, ethnicity, marital status, educational attainment, employment status, and monthly household income, and data on smoking status was collected.

### Statistical analysis

Several variables were derived from information collected using the DSHI. A dichotomous NSSI variable was created and scored as ‘yes’ for participants who endorsed ever engaging in any one of the DSHI items (i.e., lifetime NSSI). Following definitions used in previous research work [40, 41], repetitive NSSI was defined as having at least five acts of NSSI behaviours during the lifetime. Data on self-injurious behaviour that last occurred within one year was used to establish the 12-month prevalence of NSSI. For participants who endorsed multiple forms of NSSI behaviour, the age of onset of lifetime NSSI was recorded as the earliest age for any NSSI act that occurred.

Descriptive statistics were employed to describe the study sample profile, determine the prevalence of NSSI and the age of onset, and explore the types of NSSI behaviour. A chi-square test was conducted to compare gender differences in the prevalence of NSSI and types of NSSI acts. Multiple logistic regression analysis was performed to explore sociodemographic correlates of lifetime NSSI. A separate logistic regression analysis was conducted to examine behavioural and psychosocial factors associated with lifetime NSSI while controlling for sociodemographic variables. Depressive symptoms, anxiety symptoms, stress symptoms, coping strategies, resilience, perceived social support, body image concerns, smoking status, and alcohol use were treated as predictors in the regression model, while the presence of lifetime NSSI was treated as the outcome variable. To ensure that the findings were representative of the youth population in Singapore, all estimates were weighted and post-stratified for age group, gender, and ethnic distributions based on the 2022 Singapore Population Census. Statistical significance was assessed at *p* < 0.05. All analyses were performed using the Statistical Analysis Software version 9.4 (SAS Institute, Cary, NC). No missing data was encountered in this survey. Data from participants who were divorced, separated or widowed were removed from the analysis, given that this group consisted of only 37 participants (< 5% of the total sample), and meaningful inferences could not be made from their inclusion.

## Results

A total of 2600 youths participated in the study and the characteristics of the study sample are shown in Table 1.

**Table 1:** Sociodemographic profile of study sample (*N* = 2600)

		*N*	Wt % ^a^
Age group	15–19	632	18.8
	20–24	672	21.2
	25–29	634	25.4
	30–35	662	34.6
Gender	Female	1219	50.2
	Male	1381	49.8
Ethnicity	Chinese	1313	71.5
	Malay	658	16.4
	Indian	506	9.1
	Others	123	3.1
Marital status	Married/cohabiting	557	24.6
	Single	2006	74.1
	Separated/divorced/ widowed	37	1.4
Educational attainment	Primary and below	182	5.1
	Secondary school	520	16.4
	Pre-University/junior college	260	8.7
	Vocational/technical diploma	314	9.4
	Polytechnic/other diploma	681	25.5
	University and above	643	34.8
Employment status	Employed	1461	62.3
	Unemployed	166	6.4
	Economically inactive^b^	973	31.3
Monthly household income (S$) ^c^.	Below 5000	974	31.4
	5000 to 9999	859	33.8
	10,000 to 19,999	550	25.0
	20,000 and above	217	9.8

^a^Weighted percentage; percentages were weighted to be representative of the 2022 Singapore population

^b^Includes students and homemakers

^c^1 SGD is equivalent to 0.75 USD as of 21 February 2025

### Prevalence of NSSI

The lifetime and 12-month estimated prevalence of NSSI among youths in Singapore was 25.0% (95% CI: 23.1–26.8) and 6.8% (95% CI: 5.7–7.8), respectively. Significantly more females than males engaged in NSSI during their lifetime (*p* < 0.001; Table 2) and in the past one year (*p* < 0.001). 11.6% of youths engaged in repetitive NSSI and this was significantly more common among females than males (14.9% vs. 8.1%; *p* < 0.001).

**Table 2:** Prevalence rates of NSSI in overall sample and across genders

	Lifetime NSSI			12-month NSSI		
	N	Wt %	p-value	N	Wt %	p-value
Overall	680	25.0		198	6.8	
Female	393	30.2	**< 0.001**	119	8.6	**< 0.001**
Male	287	19.7		79	5.0	

Bold font indicates statistical significance

Table 3 presents the frequency of each NSSI behaviour in the overall sample and across genders and the reported age of onset for the behaviour. The most common type of NSSI behaviour among youths was cutting (13.4%), followed by severe scratching (7.9%), punching oneself (4.9%), and banging head (4.9%). Other types of NSSI behaviours, for example, slapping of oneself, punching walls/objects, snapping rubber bands on skin etc., were reported in 2.6% of youths. Significant gender differences were observed for the following types of NSSI behaviours, with more females reported engaging in them: cutting (*p* < 0.001), carving words into skin (*p* = 0.010), severe scratching (*p* < 0.001), sticking a sharp object into skin (*p* = 0.047), and preventing wounds from healing (*p* < 0.001).

**Table 3:** Frequency and age of onset by NSSI type in overall study sample and across genders

	Overall (N = 2600)		Females (N = 1219)		Males (N = 1381)			Age of onset
	N	Wt %	N	Wt %	N	Wt %	χ^2^ p-value	Mean (SD)
Cutting	364	13.4	257	19.0	107	7.7	**< 0.001**	15.1 (3.6)
Burning with cigarette	42	1.3	14	0.9	28	1.7	0.245	16.7 (2.3)
Burning with lighter or match	50	1.6	23	1.5	27	1.7	0.671	15.8 (2.4)
Carving words into skin	77	2.6	53	3.5	24	1.7	**0.010**	15.1 (3.5)
Carving pictures or marks into skin	60	2.3	33	2.8	27	1.9	0.316	16.5 (5.5)
Severe scratching	215	7.9	152	11.4	63	4.3	**< 0.001**	15.3 (4.6)
Biting	64	2.3	35	2.7	29	1.9	0.404	14.5 (4.6)
Rubbing sandpaper	13	0.5	4	0.4	9	0.6	0.573	14.0 (3.0)
Dripping acid	4	0.1	2	0.2	2	0.1	0.747	18.0 (5.3)
Using bleach to scrub the skin	3	0.1	–	–	3	0.2	–	–
Sticking sharp object into the skin	75	2.7	45	3.4	30	1.9	**0.047**	14.4 (4.5)
Rubbing glass	11	0.4	8	0.5	3	0.2	0.405	15.7 (2.9)
Breaking bones	11	0.4	5	0.4	6	0.4	0.502	16.8 (3.5)
Banging head	130	4.9	53	4.5	77	5.3	0.285	16.2 (5.7)
Punching oneself	136	4.9	61	4.6	75	5.2	0.802	16.7 (4.9)
Preventing wounds from healing	58	2.3	41	3.4	17	1.1	**< 0.001**	13.0 (5.4)
Other forms	63	2.6	37	3.3	26	1.9	**0.016**	16.3 (4.5)

Bold font indicates statistical significance

Wt % refers to weighted percentages

### Age of onset of lifetime NSSI

**Fig. 1:**
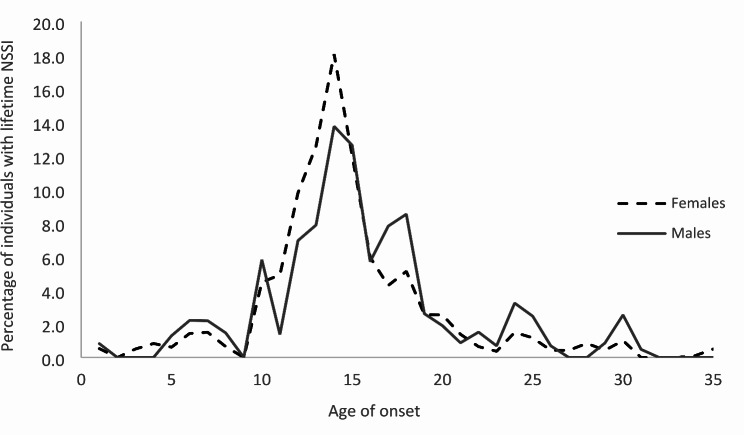
Distribution of age of onset of first NSSI episode by gender

The median age of onset for lifetime NSSI was 14 years (IQR 13–17). Figure 1 shows the distribution of age of onset of lifetime NSSI by gender. The peak age of onset occurred at around 14 years old for both females and males, with a second smaller peak observed for males at around 18 years old.

### Sociodemographic correlates of lifetime NSSI

Youths aged 15–19 (OR = 2.113, *p* = 0.002; Table 4), 20–24 (OR = 2.011, *p* < 0.001) and 25–29 (OR = 1.511, *p* = 0.014) all had significantly higher odds of lifetime NSSI than those aged 30–35 years. Gender remained significantly associated with NSSI after controlling for other sociodemographic factors; females were approximately twice more likely than males to report ever engaging in NSSI (OR = 1.875, *p* < 0.001). Educational attainment was associated with lifetime NSSI. Youths with lower educational attainment of primary and below, secondary school, and vocational/technical diploma had higher odds of reporting lifetime NSSI compared with youths with university and above educational attainment (OR = 1.589–1.990).

### Behavioural and psychosocial factors associated with lifetime NSSI

Youths with severe and extremely severe depressive symptoms (OR = 1.468, *p* = 0.030; Table 5) and anxiety symptoms (OR = 1.464, *p* = 0.013) were associated with higher odds of ever engaging in NSSI. Greater use of avoidance coping was associated with higher odds of lifetime NSSI (OR = 1.049 *p* < 0.001). Youths with body shape dissatisfaction were twice as likely (OR = 1.969, *p* < 0.001) to ever engage in NSSI compared to youths without body shape dissatisfaction. Behavioural factors including hazardous alcohol use (OR = 1.769, *p* = 0.005) and daily smoking (OR = 1.735, *p* = 0.011) were also significantly associated with higher odds of lifetime NSSI. Resilience was found to be a protective factor of lifetime NSSI; higher resilience scores were associated with lower odds of engaging in NSSI (OR = 0.989, *p* = 0.016).

**Table 4:** Behavioural and psychosocial factors associated with lifetime NSSI

		OR	95% CI		p-value
Age group	15–19	2.113	1.319	3.386	**0.002**
	20–24	2.011	1.364	2.966	**< 0.001**
	25–29	1.511	1.088	2.097	**0.014**
	30–35	Reference			
Gender	Female	1.875	1.511	2.328	**< 0.001**
	Male	Reference			
Ethnicity	Malay	1.279	0.985	1.660	0.065
	Indian	0.988	0.752	1.297	0.928
	Others	1.171	0.754	1.817	0.483
	Chinese	Reference			
Marital status^a^	Married	0.992	0.714	1.377	0.9601
	Single	Reference			
Educational attainment	Primary and below	1.989	1.142	3.464	**0.015**
	Secondary school	1.589	1.007	2.508	**0.047**
	Pre-University/junior college	1.096	0.674	1.783	0.711
	Vocational/ technical diploma	1.990	1.305	3.033	**0.001**
	Polytechnic/other diploma	1.217	0.868	1.705	0.254
	University and above	Reference			
Employment status	Unemployed	1.400	0.913	2.147	0.123
	Economically inactive	0.810	0.593	1.107	0.186
	Employed	Reference			
Monthly household income (S$)	5000 to 9999	1.061	0.812	1.386	0.663
	10,000 to 19,999	1.181	0.873	1.599	0.281
	20,000 and above	1.166	0.778	1.746	0.458
	Below 5000	Reference			

Bold font indicates statistical significance

^a^Youths who were divorced, separated or widowed (*n* = 37) were not included in the analysis due to small sample size

**Table 5:** Behavioural and psychosocial factors associated with lifetime NSSI

	OR	95% CI		*p*-value
Depressive symptoms	1.468	1.038	2.078	**0.030**
Anxiety symptoms	1.464	1.083	1.979	**0.013**
Stress symptoms	1.114	0.77	1.613	0.566
Avoidance coping	1.049	1.022	1.077	**< 0.001**
Body shape dissatisfaction	1.969	1.51	2.566	**< 0.001**
Hazardous alcohol use	1.769	1.191	2.629	**0.005**
Daily smoking	1.735	1.132	2.658	**0.011**
Problem solving coping	0.986	0.96	1.012	0.289
Resilience	0.989	0.98	0.998	**0.016**
Perceived social support	0.992	0.888	1.108	0.882

Bold font indicates statistical significance

Logistic regression adjusted for age, gender, ethnicity, marital status, educational attainment, monthly household income, and employment status

## Discussion

This study established a lifetime NSSI prevalence of 25.0% and 12-month prevalence of 6.8% among youths in Singapore, with a median age of onset of 14 years. Youths in this study engaged in various forms of self-injurious behaviours, with cutting as the most common NSSI act. Sociodemographic variables including age, gender, and educational attainment, as well as behavioural and psychosocial factors including mental health symptoms, coping strategies, body shape dissatisfaction, daily smoking, and hazardous alcohol use, were found to have significant associations with NSSI.

Consistent with findings in the literature, results from this study indicate that self-injurious behaviour is common amongst youths. The prevalence estimate of 1 in 4 youths is comparable with that reported in the literature: a meta-analysis of studies conducted between 2010 and 2021 reported an aggregate lifetime prevalence of 22.0% among adolescents [7], while another meta-analysis of studies published between 1989 and 2018 found an aggregate lifetime prevalence of 22.1% amongst children and adolescents [42]. The 12-month prevalence of NSSI (6.8%) found in this study is considerably lower than the lifetime prevalence, and this was in contrast with the meta-analyses by Xiao et al. [7] and Lim et al. [42], which found similar rates between aggregate lifetime and 12-month prevalence. However, both these studies were conducted using study samples comprising ofchildren and adolescents. This is in contrast to the present study which included older participants. It is plausible that the lower 12-month prevalence compared to the lifetime prevalence observed in this study could be attributed to older participants who had ceased engaging in NSSI, perhaps through professional intervention or learning of healthier coping methods with increasing maturity. It is an encouraging finding, given that cessation and sustained cessation of NSSI are associated with better psychological and functioning outcomes [43, 44]. While repetitive NSSI occurred less frequently (prevalence of 11.6%) amongst youths in this study, the finding from a study by Daukantaitė et al. [25] showed that both repetitive NSSI and even occasional engagement in NSSI during adolescence predicted negative mental health outcomes in young adulthood. This highlights the enduring impact of NSSI behaviours over time and underscores the importance of early detection and intervention for these behaviours in youths.

Results from the present study identified several risk factors of NSSI associated with higher odds of lifetime NSSI behaviours. Firstly, youths in the younger age group, typically those in early- and mid-adolescence. Studies in the literature have shown that NSSI generally has an early onset in adolescence and the prevalence declines from adolescence to young adulthood [10, 25]. The mean age of onset reported in this study was 15 years, with peak onset occurrence at 14 years old and a second smaller peak at 18 years old for males. These findings taken together indicate the need to focus attention on youths in the younger age group, particularly those in adolescence. These time periods coincide with secondary and post-secondary education for youths in Singapore and may represent periods of distress arising from stressors such as unsatisfactory peer relationships or poor academic performance, factors which have been found to be associated with NSSI behaviours [45, 46]. Youths might be engaging in NSSI for interpersonal reasons, such as expressing anger at others or wanting to fit in social groups [47]. Emotional distress arising from these stressors could also lead to NSSI engagement to regulate negative affect and social situations [1], particularly when youths have limited access to or lack awareness of more effective coping mechanisms. They could be engaging in NSSI to manage negative emotions or thoughts, for example to escape unwanted feelings or to use NSSI as a form of self-punishment; in such instances, NSSI serves an intrapersonal function to regulate emotions [48].

The second risk factor associated with higher odds of lifetime NSSI behaviours was being female. Females were also more likely than males to engage in certain NSSI methods such as cutting, carving words into skin, and preventing wounds from healing. Regarding gender differences in NSSI behaviours, the findings in the literature remain debatable. A meta-analysis of studies among adolescents identified female gender as a risk factor for NSSI [16]. Another recent meta-analysis by Moloney et al. [49] found the same gender effect on NSSI prevalence among adolescents in North America and Europe, but the gender difference was not significant in Asia, with a reversed pattern (i.e., higher prevalence among males) reported in some study samples. The authors proposed several reasons for this disparity including differences in gender roles, sociocultural factors such as stigma levels, and potential differences in the emotion regulation function of NSSI in Asian versus Western cultures; these factors may lead to underreporting in certain groups and thus contributing to geographical variations of NSSI prevalence by gender (see Discussion by Moloney et al.). The gender effect on NSSI may also change as a function of age, with the differences significant during mid-adolescence and less apparent in the periods before or after [50]. More studies would be needed to fully comprehend the variations in the associations between gender and NSSI in youths, and the interaction with age and geographical or potentially cultural differences.

A significant association between lifetime NSSI and lower educational attainment (i.e., primary and below, secondary school, vocational/technical diploma vs. university and above) was found, while taking into account the effects of other sociodemographic variables. To date, few studies have identified an association between education status and NSSI in youths, except for one study which found that the current education status of adolescents and young adults was associated with NSSI thoughts and behaviours, where a higher prevalence was observed in the low, middle or other education status group compared with those with high education status [51]. Both NSSI and lower educational attainment are often associated with disadvantaged social and environmental situations, such as living in an abusive environment [52, 53], which are identified risk factors of self-harm in young people [54] and may work in combination to contribute to the initiation and maintenance of NSSI behaviours. It is also plausible that youths could have lower educational attainment because of early school dropout. They may face poorer professional and social integration due to limited educational and vocational opportunities, which in turn increases their vulnerability to developing mental health issues [55] and be at risk of engaging in NSSI or other self-harm behaviours such as suicide [56].

This study found that youths having severe and extremely severe depressive and anxiety symptoms had higher odds of engaging in NSSI. The independent association between NSSI and depressive symptoms in adolescents has been well reported in previous studies [10, 17], though to a lesser extent for the association between NSSI and anxiety symptoms [23, 57]. One of the motives often cited by youths for engaging in NSSI was owing to overwhelming negative emotions and the need to reduce distress [3, 58–60], and this could explain the direct association between NSSI behaviours and depressive and anxiety symptoms. It is also plausible that the feeling of shame arising from engagement in NSSI [61] contributed to depressive and anxiety symptoms. Furthermore, repeated exposure to stigmatizing language surrounding NSSI may have compounded these negative effects [62], further harming youths with NSSI and creating a barrier to seeking help [63]. The study finding points to the importance of concurrently screening for self-injurious thoughts and behaviours alongside depressive and anxiety symptoms among youths in distress.

The association between NSSI and substance use has been well documented [64, 65]. Given the low prevalence of illicit drug use among youths in Singapore (lifetime prevalence of 2.8% among those aged 15–34 years [66]), the present study focused on examining the association between NSSI and substance use such as nicotine and alcohol. Daily smoking and hazardous use of alcohol were found in this study to be significantly associated with higher likelihood of ever engaging in NSSI. One plausible explanation for this association could be a common underlying motivation for engaging in NSSI and for hazardous consumption of alcohol and nicotine: to reduce negative or unwanted emotions [65, 67]. Youths have often reported smoking and drinking as a coping mechanism to alleviate negative emotions [68, 69], and they may have similarly turned to NSSI as a coping tool. Another possible explanation for the association could be understood from the lens of impulsivity. Smoking and alcohol use point to poor impulse control, and the link with heightened impulsivity is well supported in the literature [70–72]. Impulsivity in the form of negative urgency (i.e., taking rash actions in response to negative emotional states) has been associated with self-harming behaviours [73, 74].

In line with the literature, it was found in this study that youths who had a greater use of avoidance coping strategies were associated with higher odds of ever engaging in NSSI. Avoidance coping is considered to be maladaptive and reliance on this style of coping is associated with depressive and anxiety symptoms [75, 76] and NSSI behaviours [77, 78] in youths. Findings from this study provide further evidence to suggest that youths who lack healthy or more effective coping mechanisms are likely to turn to self-injurious behaviours to cope. In contrast to the study by Wu and Liu [79], greater use of problem-solving as a coping strategy was not significantly associated with lower odds of NSSI behaviours in this study. One possible explanation for this lack of association may be attributed to the level of psychological distress in our study sample. Elevated distress in adolescents can impair cognitive functions such as attention and memory which are critical for problem-solving [80], and rumination in depressed patients were found to contribute to poorer problem-solving solutions [81]. Youths in this study sample may be experiencing significant psychological distress and may find problem-solving strategies less effective in mitigating NSSI behaviours, thus accounting for the lack of association with NSSI behaviours observed in this study. Given that the present study has investigated two types of coping strategies, future studies may consider using instrument such as the Brief COPE [82] to explore a broader range of coping methods (e.g., denial, positive reframing) in understanding the associations with NSSI.

On the other hand, better resilience appears to be a protective factor in that it is associated with lower odds of ever engaging in NSSI among youths in this study. Several studies have identified a similar association between resilience and NSSI behaviours; these studies generally showed that compared with individuals without NSSI behaviours, those with current and past NSSI behaviours had lower levels of resilience, and those currently engaging in NSSI acts had the lowest resilience level [83–85]. Resilience, broadly defined as the capacity of an individual to successfully adapt and produce positive outcomes in the face of adversities [86], is a psychological resource that individuals can tap onto to cope with life stressors. Resilience in itself encompasses individual assets (e.g., self-efficacy and competence) and resources (e.g., parental support and adult mentoring; [87]), which are factors that can indirectly contribute to reduced NSSI behaviours through the use of more adaptive coping strategies.

Given that resilience can be strengthened through interventions [88], schools and individuals working with youths should consider incorporating resilience-building programs into their curricula or in their interactions with youths. In Singapore, there are existing mental wellness and resilience programs in schools to train students on topics such as emotion regulation, problem solving and coping strategies to build up their social-emotional competencies [89]. Resources are also available for parents to understand how they can support their children in their social and emotional learning, for instance by interacting with them in a manner that can foster resilience [90]. It may be useful to complement these existing programs and resources with enhanced content of resilience-building with the intention of reducing NSSI and other negative behavioural outcomes that are often associated with mental health distress.

There are some limitations to consider when interpreting the findings from this study. Firstly, causal associations between the variables cannot be determined, given that cross-sectional data were collected. Next, participants may have been reluctant to share their self-injurious experiences due to stigma, leading to an under-reporting of the prevalence. However, the effect was minimised given that the entire survey was self-administered, participants were informed and reassured of their confidentiality and the anonymity of the study data collected, and they were also given the option to skip questions in the section assessing NSSI behaviour should they feel uncomfortable answering it. Another limitation is that this study relied on self-reported data which is inherently subjective and susceptible to recall bias, potentially leading to less reliable prevalence estimates. Future studies could enhance data accuracy by incorporating objective measures, such as data from medical records, or corroborating self-reported data with clinical assessments. Lastly, given that NSSI rates have been found to differ throughout the COVID-19 period [91], it is worthwhile to note that the present study was conducted in the post-pandemic era. Data collection for this study began in October 2022, sometime after the acute phases of the pandemic in 2020 and 2021 when rates of infection were high and with many social restrictions in place (e.g., limit on group size for gatherings). It is nonetheless possible that potential long-term effects of COVID-19 on mental health [92, 93] had an influence on NSSI behaviour that was not investigated in the present study. Notwithstanding the above limitations, the strength of the study includes the large sample size that is representative of the youth population in Singapore, and in addition, efforts were made to invite youths from diverse backgrounds or hard-to-reach groups to participate in the study, e.g., youths in National Service, youths not in education, employment, or training. Thus, this allows the results from the study to be generalizable to the youth population in Singapore. A checklist of NSSI behaviours using the DSHI was utilised in this study which facilitated recall for participants and thus provided more accurate prevalence estimates as compared with single yes or no question [6]. Furthermore, stringent quality control measures were taken and regular monitoring of study records was implemented to ensure the quality of data. This includes an extensive training of field interviewers on various aspects of the research study including consent taking and questionnaires administered in the survey, and also detailed data verification was done on 20% of cases completed by each interviewer.

Taken together with findings from previous research, results from this study provide several valuable recommendations and considerations that could be key to reducing NSSI behaviours in youths. Firstly, given that the onset and peak of NSSI occurrences in youths overlap with schooling years, an important implication would be to equip school personnel including teachers, counsellors and administrators with the knowledge and skills to detect and respond appropriately to self-injury in students. School personnel have reported feeling inadequate in providing support and in managing self-injurious behaviour among students [94–96] and this can act as a barrier for youths to report and seek help for self-injurious behaviour [97]. Furthermore, youths are likely to confide in their peers and disclose their NSSI behaviours [98], leading to a possible contagion effect where exposure to peers’ NSSI behaviours influences adolescents’ own behaviours [99]. Peers may also be ill-equipped to respond appropriately to disclosure which can perpetuate negative feelings and act as a barrier to help-seeking [100]. Interventions at a broader, school-based level to address and tackle NSSI among youths are thus needed, and these should not just focus on the youths themselves but also on training school staff and faculty. Aside from school peers and personnel, it is also crucial to involve parents in the process given that a supportive family environment is beneficial to the help-seeking process and treatment which can lead to a reduction in NSSI behaviours [101].

Next, preventive programs can be developed to reduce the onset of self-injurious behaviours. Such specialized programs could include various psychoeducational components aimed at enhancing resilience in youths or teaching healthier coping strategies, specifically more effective ways of regulating emotions (e.g., the DUDE (Du und deine Emotionen; i.e., You and your emotions) program in Germany [102]). Based on the findings of this study, NSSI preventive programs and interventions could be targeted at the more vulnerable youth groups as identified in this study, which include those who are in early- and mid-adolescence, females, youths with lower educational attainment, youths with severe mental health distress, and youths who smoke daily or those who consume alcohol in hazardous amount. Several universal prevention programs for NSSI have demonstrated promising results, however, more work is needed to develop evidence-based approaches and subsequently evaluate them [103].

Lastly, it is pertinent to improve awareness and knowledge of NSSI on a broader societal level. Often because of the shame and stigma associated with it, NSSI tends to remain undiscovered until it requires professional intervention, and even then, youths may choose to remain reticent [104, 105].Professionals and non-professionals have displayed perceived unhelpful responses to NSSI behaviours (e.g., related to themes of ‘disapproval or judgment’ and ‘avoidance and inaction’) which discouraged individuals from help-seeking [106]. Thus, there is a need to facilitate open communications about NSSI and foster an understanding and non-judgemental environment in which youths feel safe to seek professional help, and also one in which their peers are prepared to provide support and direct them to trusted-adults.

In conclusion, the phenomenon of self-injurious behaviours among youths is more common than it seems. Screening of NSSI behaviours amongst school-aged youths and imparting adaptive emotional regulation or coping strategies could contribute to reducing NSSI thoughts and behaviours. School personnel and those working with youths are well-positioned to offer support to youths, particularly in inculcating resilience and introducing healthy coping strategies.

## Data Availability

Data are not available for online access, however, readers who wish to gain access to the data can write to the corresponding author with their requests. Access can be granted subject to the institutional review board (IRB) and the research collaborative agreement guidelines.
